# Assessment of the efficacy and safety of tocilizumab in patients over 80 years old with giant cell arteritis

**DOI:** 10.1186/s13075-021-02529-4

**Published:** 2021-05-19

**Authors:** Hubert de Boysson, Maelle Le Besnerais, Félix Blaison, Aurélie Daumas, Pierre-André Jarrot, François Perrin, Nathalie Tieulié, Alexandre Maria, Pierre Duffau, Bruno Gombert, Maxime Samson, Olivier Espitia, Marc Lambert, Arsène Mékinian, Achille Aouba, for the French Study Group for Large Vessel Vasculitis (GEFA)

**Affiliations:** 1 Department of Internal Medicine, Caen University Hospital, Avenue de la Côte de Nacre, 14000 Caen, France; 2 Normandy University, Unicaen, Caen, France; 3 Department of Internal Medicine, Rouen University Hospital, Dijon, France; 4 Department of Internal Medicine, Bordeaux University Hospital, Bordeaux, France; 5 Department of Internal Medical and Clinical Immunology, Conception University Hospital, Marseille, France; 6 Department of Internal Medicine, Saint-Nazaire Hospital, Saint-Nazaire, France; 7 Department of Rheumatology, Nice University Hospital, Nice, France; 8 Department of Internal Medicine, Montpellier University Hospital, Montpellier, France; 9 Department of Rheumatology, La Rochelle Hospital, La Rochelle, France; 10 Department of Internal Medicine, Dijon University Hospital, Dijon, France; 11 Department of Internal Medicine, Nantes University Hospital, Nantes, France; 12 Department of Internal Medicine, Lille University Hospital, Lille, France; 13 Department of Internal Medicine, Saint-Antoine Hospital, Paris, France

**Keywords:** Giant cell arteritis, Tocilizumab, Elderly, Old patients, Efficacy, Safety

## Abstract

**Objective:**

To assess the efficacy and tolerance of tocilizumab (TCZ) in giant cell arteritis (GCA) patients over 80.

**Method:**

GCA patients over 80 years old from the French Study Group for Large Vessel Vasculitis register who received TCZ were analyzed.

**Results:**

Twenty-one GCA patients (median age 84 [81–90] years old, including nine over 85) received TCZ for the following nonexclusive reasons: glucocorticoid (GC)-sparing effect in 14, relapsing disease in 8, disease severity in 4, and/or failure of another immunosuppressant in 4. TCZ was introduced with GCs at diagnosis in 6 patients and at 8 [3–37] months after GC initiation in 15 others. After a median delay of 8 [2–21] months post-TCZ introduction, 14 (67%) patients were able to definitively stop GCs, including 6 who were GC-dependent before TCZ. At the last follow-up (median 20 [3–48] months), 11 (52%) patients had definitively stopped TCZ, and 2 additional patients had stopped but relapsed and resumed TCZ.

Seven (33%) patients experienced 11 adverse events: hypercholesterolemia in 4 patients; infections, i.e., pyelonephritis, bronchitis, and fatal septic shock associated with mesenteric infarction following planned surgery (GCs were stopped for 1 year and TCZ infusions for 2 months), respectively, in 3 patients; moderate thrombocytopenia and moderate neutropenia in 2 patients; and a 5-fold increase in transaminase levels in another that improved after TCZ dose reduction.

**Conclusion:**

TCZ remains a valuable GC-sparing option in the oldest GCA patients with an interesting risk-benefit ratio. Mild-to-moderate adverse events were observed in one-third of patients.

## Introduction

Giant cell arteritis (GCA) is a large- and medium-sized vasculitis occurring mainly in patients over 50 years old and is the most frequent systemic vasculitis in elderly patients from Western countries. Epidemiological data show that the incidence of GCA increases with advancing age, and some studies have suggested that the mean age of GCA onset is still increasing, with a maximal incidence between 70 and 80 years old [1–5]. However, patients over 80 years old are common and represent a subgroup with increased frailty, including age-related immunodeficiency, modification of drug metabolism, increased cardiovascular risk factors, and reduced muscular autonomy [6]. This frailty is worsened by the long-term use of glucocorticoids (GCs), which remain the cornerstone of GCA treatment. Taken together, these findings raise the question of whether different therapeutic strategies could be used for elderly individuals, especially to reduce GC exposure while keeping the disease under control. Little information exists on the use of targeted therapies in elderly patients. To date, immunosuppressants, especially methotrexate, have been advised for patients with relapsing disease or for whom GCs should be spared because of toxicity. More recently, tocilizumab (TCZ), a monoclonal antibody targeting the IL-6 receptor, has been approved for GCA. TCZ has shown effectiveness in achieving disease remission and a good GC-sparing effect with an acceptable tolerance profile in a GCA population [7]. However, the mean age of the population in whom the treatment was validated was 69.5 ± 8.5 years, and no subgroup analysis focused on the efficacy and tolerance of TCZ in the oldest patients. In the present study, we aimed to assess the efficacy and tolerance of TCZ in patients over 80 years included in a French register of GCA patients treated with TCZ.

## Patients and methods

### Patients

In January 2019, physicians belonging to the French Study Group for Large Vessel Vasculitis (GEFA) were asked to include in a register their patients who received TCZ for the treatment of GCA for at least 2 months. GCA diagnosis was retained if the patient satisfied at least 3 criteria from the American College of Rheumatology, showed a positive temporal artery biopsy (TAB), or demonstrated large-vessel vasculitis on imaging. A standardized electronic form was sent to each physician who declared a patient. Anonymized data were thus collected in a centralized database.

In December 2020, 186 patients were included in the register, including 21 (11%) patients over 80 years old.

The Caen Ethical Board approved the study (CLERS ID265).

### Data collection and definition

The data collection included demographics, cardiovascular risk factors (hypertension, hypercholesterolemia, diabetes mellitus, tobacco use), GCA-related cranial symptoms (headaches, scalp tenderness, jaw claudication, temporal artery abnormality, visual signs), polymyalgia rheumatica (PMR), limb claudication, fever, weight loss, acute phase reactants (erythrocyte sedimentation rate [ESR], C-reactive protein [CRP]), the results of histology (TAB or another vascular sample if available), the results of imaging, treatment regimens, and outcomes.

Regarding GC management, we retrieved the date of introduction, initial dose, dose at TCZ initiation and discontinuation, and duration of treatment. When GC tapering was not possible under a certain dose because of relapses, we qualified the disease as GC-dependent.

Regarding TCZ management, we noted the delay of introduction after GCs, duration of treatment, and route used (intravenous [IV] or subcutaneous [SC]). The indication for TCZ was noted among the four possible nonexclusive following criteria: GC-sparing effect, relapsing disease, severe GCA involvement (the severity of GCA was left to the physician’s judgment), and failure of a previous immunosuppressant other than GCs.

Regarding TCZ tolerance, the following side effects were screened: infections, forms of cytopenia, hepatic or digestive toxicity, dyslipidemia requiring a specific medical treatment, or allergy. The Common Toxicity Criteria for Adverse Events (CTCAE) was used to grade the adverse events (scaling from 1 to 5), with the 5th grade corresponding to death [8].

### Statistical analysis

Categorical variables are expressed as numbers (%), and quantitative variables are expressed as medians [range].

## Results

The characteristics of the 21 patients are shown in Table 1. Nine (43%) patients were ≥85 years old at diagnosis, and 76% of the included patients were women. Among the patients, one had myelodysplasia with refractory anemia, and two were known to have colonic diverticulosis. The treatments administered to the patients are indicated in Table 2. GCs were introduced at 0.8 [0.6–1.1] mg/kg/day. The intravenous method was used to administer TCZ to 14 patients, and the subcutaneous method was used for the other 7.

**Table 1:** Baseline characteristics of patients with giant-cell arteritis over 80 years old who received tocilizumab

Characteristics	Patients (n = 21)
**Demographics**	
Age	84 [81–90]
Female sex	16 (76)
**Past medical history**	
Number of cardiovascular risk factors (except age and sex)	1 [0–3]
Hypertension	11 (52)
Tobacco use	2 (10)
Diabetes mellitus	4 (19)
Hypercholesterolemia	6 (29)
Overweight	2 (10)
Coronary disease	4 (19)
Previous stroke	2 (10)
Diverticulosis	2 (10)
Osteoporotic fractures	2 (10)
Myelodysplasia	1 (5)
**Clinical manifestations**	
ACR criteria	4 [3–5]
Weight loss	15 (71)
Fever	5 (24)
Headaches	17 (81)
Jaw claudication	12 (57)
Abnormal temporal artery	7 (33)
Scalp tenderness	7 (33)
Ophthalmologic involvement	7 (33)
Polymyalgia rheumatica	9 (43)
Limb claudication	1 (5)
**Laboratory parameters**	
Erythrocyte sedimentation rate, in mm	82 [25–138]
C-reactive protein, in mg/l	90 [16–224]
Hemoglobin level, g/dl	10.3 [9.2–15.1]
**Positive histology**	11/16 (69)
**Large-vessel vasculitis**^a^	7/16 (44)
At GCA diagnosis	4/13 (31)
During follow-up	3/3 (100)

Values are numbers (%) or medians [range]. *CV*, cardiovascular; *ACR*, American College of Rheumatology; *GCA*, giant-cell arteritis

^a^In patients who underwent large-vessel imaging

**Table 2:** Treatment and outcomes of patients with giant-cell arteritis over 80 years old who received tocilizumab

Characteristics	Patients (n = 21)
**Total follow-up, in months**	20 [3–48]
**Antiplatelets**	18 (86)
**Glucocorticoid use**	
Initial dose, mg/kg	0.8 [0.6–1.1]
Duration of intake in all patients^a^, in months	14 [2–48]
Number of patients who stopped GCs after TCZ introduction	14 (67)
Delay to stop GCs after TCZ introduction	8 [2–21]
**Tocilizumab use**	
Introduction at GCA diagnosis	6 (29)
Introduction during the FU	15 (71)
Delay of introduction in patients with TCZ introduction at FU	8 [3–37]
First-line immunosuppressant	17 (81)
Second-line immunosuppressant	4 (19)
Number of patients who discontinued TCZ at last FU	11 (52)
Duration of TCZ use in all patients, in months	7 [3–28]
Duration of TCZ in patients who stopped it, in months	8 [3–28]
**Number of patients with a control of large-vessel imaging**	3
Improvement	2
No improvement	1
**Relapses before and after TCZ introduction**	
Number of patients who relapsed	11 (52)
Total number of relapses	19
Delay of the first relapse, in months	5 [2–19]
Number of relapses before TCZ	15
Under TCZ	2
After TCZ discontinuation	2
Delay of relapse after TCZ discontinuation, in months	1 and 13
**Death**	4 (19)

Values are numbers (%) or medians [range]. *GCs*, glucocorticoids; *FU*, follow-up; *TCZ*, tocilizumab

^a^From GC introduction to stop or to the last follow-up visit if GCs are ongoing

In 6 (29%) patients, TCZ was introduced with GCs (median initial dose 60 [30–70] mg/day) at GCA diagnosis to obtain a fast GC-sparing effect in 4 (metabolic and cardiovascular indication in 2, psychiatric indication in 2) or because of GCA-related severity in 2 (bilateral acute anterior ischemic optic neuropathy (AION) in one, large-vessel vasculitis of the 4 limbs in another). In these 6 patients, after a median follow-up of 12 [4–31] months, GC doses were tapered to 0 [0–10] mg/day, including four patients who discontinued GCs 2 to 8 months after initiation and did not relapse thereafter. Two patients had stopped TCZ after 3 months of use and did not relapse. None of these 6 patients experienced any cardiovascular event or psychiatric decompensation after treatment introduction. The patient with bilateral AION maintained important visual sequelae, and the other with peripheral large-vessel vasculitis showed improvement in clinical and imaging parameters. Only one of the 6 patients exhibited clinical relapse (headaches and polymyalgia rheumatica) in the 8th month under GC and TCZ therapy. An increase in GC dosage controlled the disease.

In the 15 (71%) remaining patients, TCZ was introduced at a median delay of 8 [3–37] months after GC initiation for the nonexclusive following reasons: GC-sparing effect in 10; relapsing disease in 8; disease severity in 2, including large-vessel vasculitis affecting the carotids with a stroke in one, relapse with AION in the last; and/or failure of a previous immunosuppressant line in 4, including methotrexate in 3 and anakinra in 1. The nonexclusive GC-related toxicities in the 10 patients in whom a sparing effect was required were neuropsychiatric deterioration in 7, amyotrophy and loss of autonomy in 4, diabetes instability in 2, symptomatic arterial hypertension in 1, and severe glaucoma in 1. The median GC dose at TCZ introduction was 20 [5–60] mg/day, which was decreased after a median of 10 [3–36] months to 0 [0–25] mg/day. Ten of the 15 patients were able to discontinue GCs after 8 [4–21] months, including 6 who were GC-dependent before TCZ. Moreover, 9/15 had stopped TCZ after a median duration of 10 [4–33] months. Altogether, 3/15 patients relapsed once TCZ was introduced. One relapse occurred during TCZ treatment with a resumption of headaches and polymyalgia rheumatica signs. The other two corresponded to clinical and biological resumption of GCA signs 1 and 13 months after TCZ discontinuation. Both patients were retreated with TCZ and exhibited new clinical remission.

Four (19%) patients in the whole cohort died after 18 [4–47] months: 2 from cardiovascular events (multiple strokes in the first, mesenteric infarction in the other); another from renal cancer while under GC and TCZ; and the last following mechanic fall complications. GCA was not directly responsible for death in these four patients.

Safety information regarding TCZ is indicated in Table 3. A total of 7 (33%) patients experienced 11 adverse events, the most common being hypercholesterolemia observed in 4 patients. Serious adverse events with ≥ grade 3 toxicities were observed in 4 (19%) patients. Intravenous administration was used in 5/7 patients. Three patients experienced infections, including 2 who were receiving concomitant GCs. Patient 6 died from septic shock and a mesenteric infarction occurring 2 days after a programmed cholecystectomy. GCs were stopped for 1 year, and TCZ infusions were temporally suspended for the 2 months prior to the surgery. No patient with diverticulosis showed complications, nor did the patient with myelodysplasia. Two cytopenias were noted: thrombopenia at 75 giga/l and neutropenia at 900/mm^3^. Finally, a 5-fold increase in transaminase levels was observed in a patient and improved with a reduction in TCZ dose from 8 to 4 mg/kg, without any GCA relapse.

**Table 3:** Adverse events and outcomes observed in patients once TCZ started for GCA

Adverse event	Months after TCZ introduction	Dose of GC at AE time	GC duration (months)	CTCAE grade	Treatment/evolution
**Patient 1**					
Hypercholesterolemia	4	10 mg	4	2	No treatment, stable
**Patient 2**					
Hypercholesterolemia	8	10 mg	8	3	Statin, improvement
**Patient 3**					
Hypercholesterolemia	3	0	–	2	No treatment, stable
**Patient 4**					
Pyelonephritis	5	10 mg	9	3	IV antibiotics-TCZ shifted 1 month later
Thrombopenia	2	15 mg	6	1 (75 G/l)	No treatment, stable
**Patient 5**					
Bronchitis	9	6 mg	13	2	Oral antibiotics, healed
Neutropenia	7	8 mg	11	3 (900/mm^3^)	No treatment, stable
Hepatic cytolysis	7	8 mg	11	3 (5N)	Reduction TCZ to 4 mg/kg, correction of cytolysis
**Patient 6**					
Septic shock^a^	20	0	–	5	Death
Mesenteric infarction^a^	20	0	–	5	
**Patient 7**					
Hypercholesterolemia	9	0	–	2	No treatment, stable

Values are numbers (%). *TCZ*, tocilizumab; *AE*, adverse events; *IV*, intravenous; *G*, giga; *N*, normal; *GC*, glucocorticoid; *CTCAE*, Common Toxicity Criteria for Adverse Events

^a^Occurred post-surgery for a programmed cholecystectomy. TCZ was temporally stopped for 2 months

## Discussion

Our study suggests that TCZ can be used with a good tolerance profile in elderly GCA patients. Although GC remains the cornerstone of the treatment and is sufficient for most patients, some circumstances can require an adjunctive immunosuppressant to achieve disease remission or to decrease GC use more quickly. Tocilizumab was recently included among the few therapeutic options available, in addition to methotrexate, for patients with relapsing GCA or those for whom a GC-sparing effect is needed [9]. Based on our results, two main points should be highlighted and discussed.

First, in accordance with the main studies dealing with TCZ, GC doses can be rapidly decreased in patients receiving TCZ. In France, current guidelines recommend using GCs for 18–24 months [10]. However, little information exists on the morbidity of such prolonged GC treatment in elderly people, in whom GC toxicity on metabolism, the cardiovascular system, muscle, or bone can be life-threatening. A reduction in GC exposure in this subset of patients should be a priority, and TCZ appears to be an option. In our study, two-thirds of patients were able to stop GC use after a median delay of 8 months, including some patients with GC-dependent disease. This result is concordant with published studies that reported sustained remission in 54 to 70% of patients, including mainly patients <80 [7, 11]. Some future studies and consensus are needed to determine whether different strategies should be proposed in elderly patients, as suggested for systemic necrotizing vasculitides [12].

Second, our study indicated that serious adverse events were limited, affecting 19% of our patients. We did not observe an over-representation of serious adverse events when compared to other published studies. In the patients of the Giacta trial who received TCZ, serious adverse events were reported in 15% of patients, mainly infections. Serious infections, grade 3 neutropenia, and transaminase elevations were observed in 7%, 4%, and 2% of patients, respectively [7]. In the Spanish study from Calderón-Goercke et al. including 134 patients in a real-life setting, serious adverse events were observed in 23.9%, mainly infections [11]. In the two aforementioned studies, TCZ was discontinued due to side effects in 6% and 12.7% of the Giacta and the Spanish cohort, respectively. Of note, no gastrointestinal perforations were reported in either study [7, 11]. Three of our patients developed infective toxicities, which are also common in patients treated with GCs alone [13, 14]. One of our patients, however, died from infection and mesenteric infarction, and the role of the underlying immunodepression cannot be excluded. Other TCZ toxicities observed in our study were common and not serious. Interestingly, no signal emerged from patients with previous diverticulosis or myelodysplasia.

Although our work demonstrates the availability of TCZ in therapeutic strategies for elderly GCA patients, the relatively small sample size, retrospective design, and short follow-up time should be acknowledged. Our patients over 80 were selected and might differ from other GCA patients regarding their baseline characteristics and outcomes. However, we believe that a description of TCZ use in this population remains useful in clinical practice. Regarding safety, our small sample might limit the capture of other adverse events. However, the comparison with larger cohorts was reassuring and indicates that the side effects we observed were representative of described tolerance profiles.

## Conclusion

In conclusion, this study highlights that tocilizumab can be an option to reduce GC exposure in GCA patients over 80 years old. Mild-to-moderate adverse events are observed in one-third of patients and thus require constant vigilance.

## Data Availability

Data are available to request.

## References

[1] Bengtsson BA, Malmvall BE (1981). The epidemiology of giant cell arteritis including temporal arteritis and polymyalgia rheumatica. Incidences of different clinical presentations and eye complications. Arthritis Rheum.

[2] Gonzalez-Gay MA, Miranda-Filloy JA, Lopez-Diaz MJ, Perez-Alvarez R, Gonzalez-Juanatey C, Sanchez-Andrade A, Martin J, Llorca J (2007). Giant cell arteritis in northwestern Spain: a 25-year epidemiologic study. Medicine (Baltimore).

[3] Hunder GG (2002). Epidemiology of giant-cell arteritis. Cleve Clin J Med.

[4] Kermani TA, Schafer VS, Crowson CS, Hunder GG, Gabriel SE, Matteson EL, Warrington KJ (2010). Increase in age at onset of giant cell arteritis: a population-based study. Ann Rheum Dis.

[5] Lopez-Diaz MJ, Llorca J, Gonzalez-Juanatey C, Pena-Sagredo JL, Martin J, Gonzalez-Gay MA (2008). Implication of the age in the clinical spectrum of giant cell arteritis. Clin Exp Rheumatol.

[6] Liozon E, Delmas C, Dumonteil S, Dumont A, Gondran G, Bezanahary H, Aouba A, Fauchais AL, Ly KH, de Boysson H (2019). Features and prognosis of giant cell arteritis in patients over 85 years of age: a case-control study. Semin Arthritis Rheum.

[7] Stone JH, Klearman M, Collinson N (2017). Trial of tocilizumab in giant-cell arteritis. N Engl J Med.

[8] National Cancer Institute. Common terminology criteria for adverse events and common toxicity criteria. URL: http://ctep.cancer.gov/protocoldevelopment/electronic_applications/ctc.htm. Accessed Mar 2021.

[9] Hellmich B, Agueda A, Monti S, Buttgereit F, de Boysson H, Brouwer E, Cassie R, Cid MC, Dasgupta B, Dejaco C, Hatemi G, Hollinger N, Mahr A, Mollan SP, Mukhtyar C, Ponte C, Salvarani C, Sivakumar R, Tian X, Tomasson G, Turesson C, Schmidt W, Villiger PM, Watts R, Young C, Luqmani RA (2020). 2018 update of the EULAR recommendations for the management of large vessel vasculitis. Ann Rheum Dis.

[10] Bienvenu B, Ly KH, Lambert M, Agard C, Andre M, Benhamou Y (2016). Management of giant cell arteritis: recommendations of the French study group for large vessel vasculitis (GEFA). Rev Med Interne.

[11] Calderón-Goercke M, Castañeda S, Aldasoro V, Villa I, Prieto-Peña D, Atienza-Mateo B (2020). Tocilizumab in giant cell arteritis: differences between the GiACTA trial and a multicentre series of patients from the clinical practice. Clin Exp Rheumatol.

[12] Pagnoux C, Quemeneur T, Ninet J, Diot E, Kyndt X, de Wazieres B (2015). Treatment of systemic necrotizing vasculitides in patients aged sixty-five years or older: results of a multicenter, open-label, randomized controlled trial of corticosteroid and cyclophosphamide-based induction therapy. Arthritis Rheumatol.

[13] Schmidt J, Smail A, Roche B, Gay P, Salle V, Pellet H, Duhaut P (2016). Incidence of severe infections and infection-related mortality during the course of giant cell arteritis: a multicenter, prospective, double-cohort study. Arthritis Rheumatol.

[14] Wu J, Keeley A, Mallen C, Morgan AW, Pujades-Rodriguez M (2019). Incidence of infections associated with oral glucocorticoid dose in people diagnosed with polymyalgia rheumatica or giant cell arteritis: a cohort study in England. CMAJ.

